# Supplementary material for: Widespread homogenization of plant communities in the Anthropocene
Source: Nat Commun. 2021 Dec 6;12:6983. doi: 10.1038/s41467-021-27186-8 (PMC8648934; doi:10.1038/s41467-021-27186-8)
Supplement: Supplementary file 1 — Supplementary information [file 41467_2021_27186_MOESM1_ESM.pdf]

Supplementary Information

Widespread homogenization of plant communities in the Anthropocene

Daru et al.

*Correspondence to: Barnabas H. Daru (barnabas.daru@tamucc.edu), T. Jonathan Davies (j.davies@ubc.ca) and Charles C. Davis (cdavis@oeb.harvard.edu)

Contents:

Supplementary Figures 1 to 5

Supplementary Tables 1 to 3

Supplementary Methods

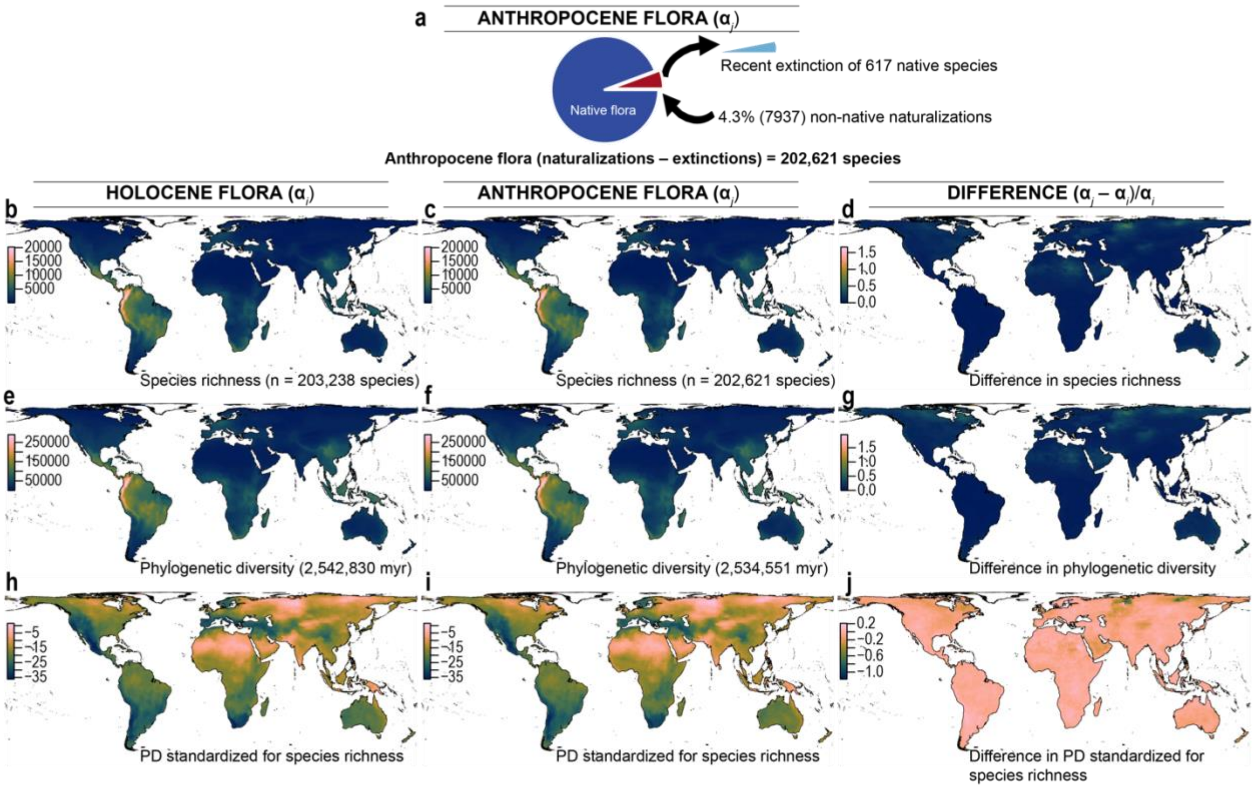

Supplementary Figure 1 | Temporal and spatial changes in α-diversity across plant communities in the Anthropocene based on recent plant extinctions and naturalisations (*best case* scenario) at a spatial resolution of 50 km × 50 km. Left panel shows the Holocene flora, middle the Anthropocene flora (based on recent extinctions and naturalisations) and right panel differences between Holocene and Anthropocene floras. (**a**) Schematic of the Anthropocene flora showing recent extinctions replaced by non-native naturalisations. (**b**), (**c**), (**d**) Spatial and temporal changes in species (α) diversity. (**e**), (**f**), (**g**) Spatial and temporal changes in observed phylogenetic (α) diversity. (**h**), (**i**), (**j**) Spatial and temporal changes in phylogenetic (α) diversity standardized for species richness (phylogenetic tip shuffling 1000 times). Species diversity was calculated as the numbers of species within 50 km × 50 km grid cells. Phylogenetic diversity (PD) was calculated in million years (myr) as the sum of all phylogenetic branch lengths for the set of species within each grid cell. Species richness was corrected for by calculating the standardized effective size of phylogenetic (α) diversity based on 1000 randomizations. Maps are in Behrmann equal-area projection.

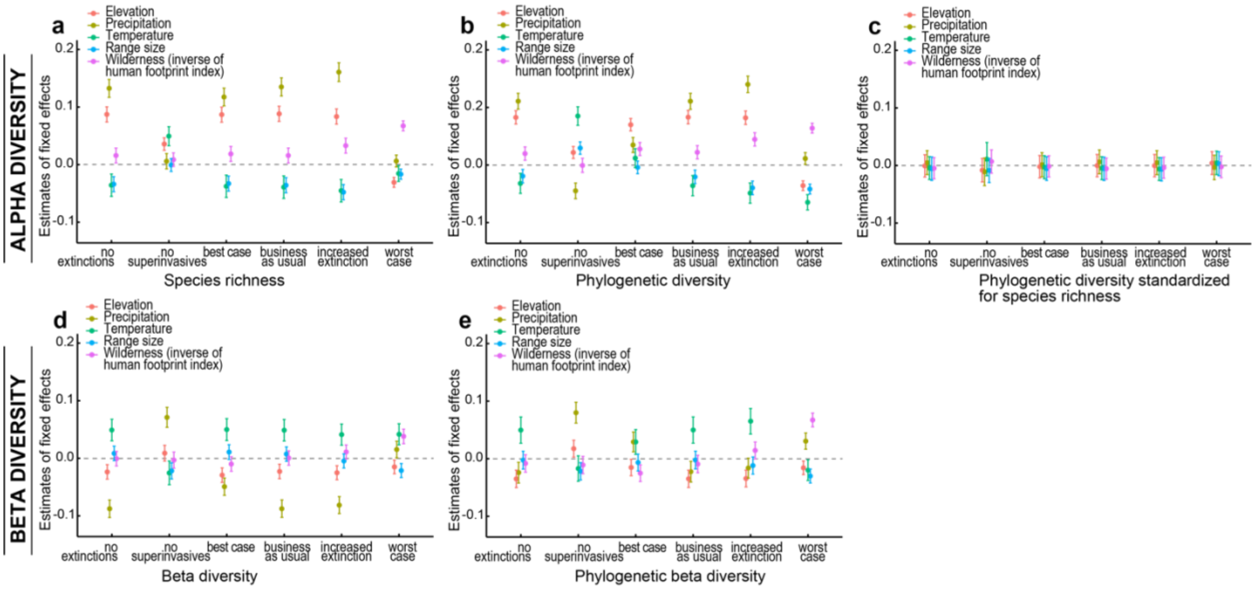

Supplementary Figure 2 | The estimates and 95% confidence intervals for the fixed effects predicted from the mixed-effects models of difference in alpha and beta diversity with all predictors (ecological, evolutionary and anthropogenic variables, spatial autocovariate, plus random effects of level 3 of TDWG biomes) across varying scenarios of extinction including naturalisations only. a, Differences in species richness with all predictors between Holocene and Anthropocene floras across varying future scenarios of extinction including naturalisations only (n = 11,341 grid cells). **b,** Differences in phylogenetic diversity between Holocene and Anthropocene floras across varying future scenarios of extinction including naturalisations only (n = 11,341 grid cells). **c,** Differences in phylogenetic diversity standardized for species richness between Holocene and Anthropocene floras across varying future scenarios of extinction including naturalisations only (n = 11,341 grid cells). **d,** Differences in beta diversity between Holocene and Anthropocene floras across varying future scenarios of extinction including naturalisations only (n = 11,341 grid cells). **e,** Differences in phylogenetic beta diversity between Holocene and Anthropocene floras across varying future scenarios of extinction including naturalisations only (n = 11,341 grid cells). These models indicate that shifts towards increasing homogeneity are most pronounced in regions of high elevation and increased wilderness. Significance was assessed by comparing likelihoods of the fitted objects. All the data are presented as estimated values ± 95% confidence intervals. Source data are provided as a Source Data file.

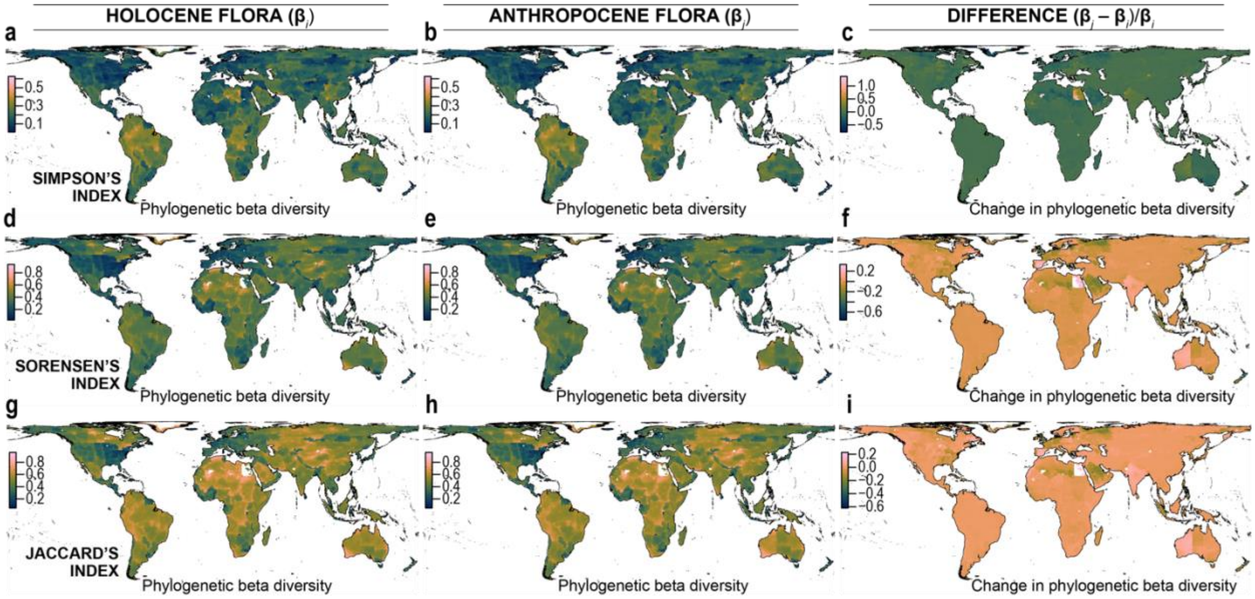

Supplementary Figure 3 | Spatial and temporal changes in phylogenetic compositional turnover (β- diversity) between the Holocene (pre-Columbian) and Anthropocene epochs (*best case scenario*) for different dissimilarity metrics. Phylogenetic turnover (β-diversity) in the Holocene (205,456 species) (left panel). Phylogenetic turnover in the Anthropocene based on naturalisations of 10,318 species, and recent extinction of 1065 species (middle panel). Differences in phylogenetic turnover (β-diversity) between the Holocene and Anthropocene epochs (right panel). Comparison is shown for Simpson's index (A, B, C); Sorensen's index (D, E, F); and Jaccard's index (G, H, I). Phylogenetic turnover was calculated using Simpson's metric of beta diversity between 100 km × 100 km grid cells within biomes as recognized by the Biodiversity Information Standards (also known as the Taxonomic Databases Working Group). The maps are in Behrmann projection.

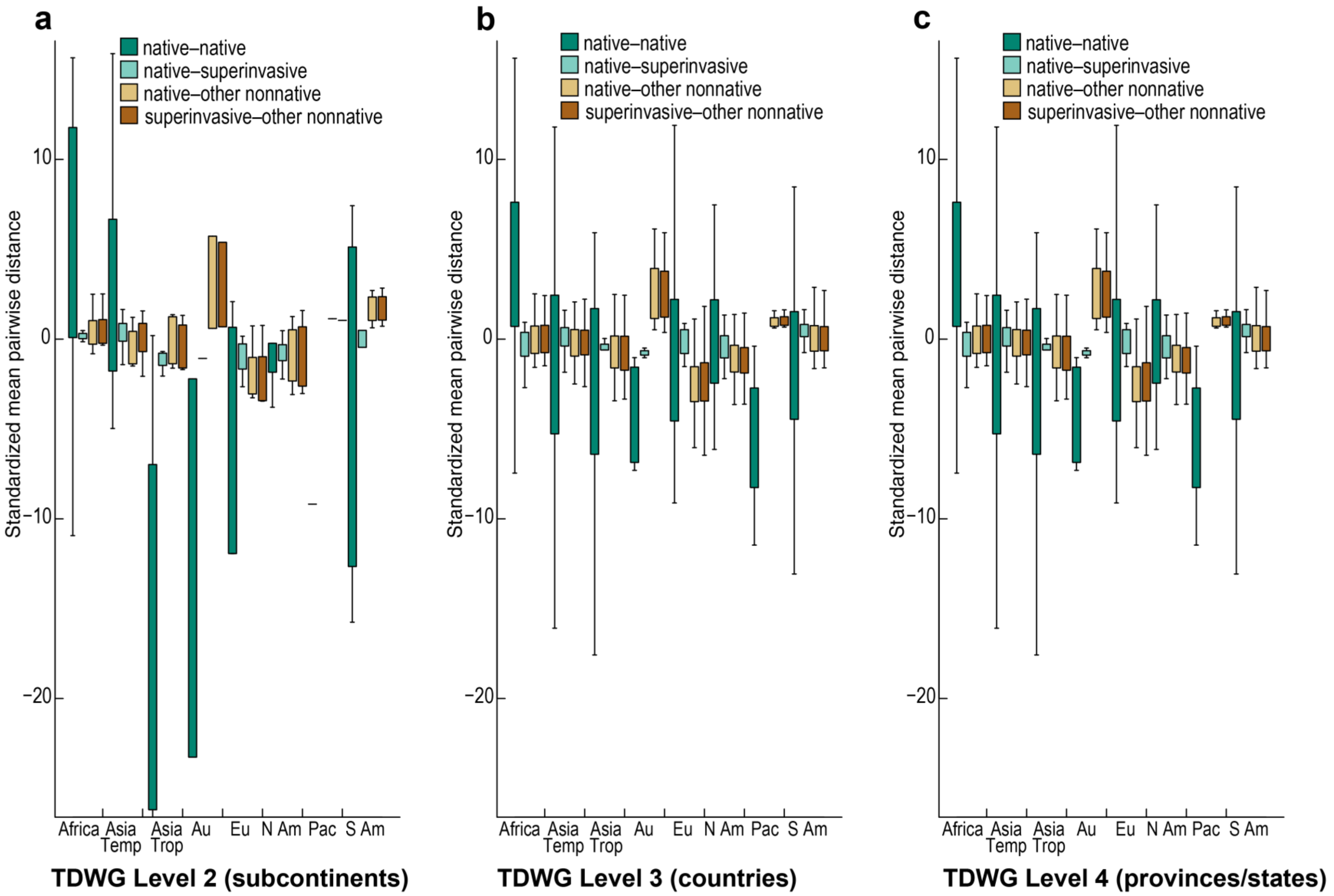
a
b
c
native–native
native–superinvasive
native–other nonnative
superinvasive–other nonnative
Standardized mean pairwise distance
10
0
−10
−20
Africa
Asia Temp
Asia Trop
Au
Eu
N Am
Pac
S Am
TDWG Level 2 (subcontinents)
TDWG Level 3 (countries)
TDWG Level 4 (provinces/states)

Supplementary Figure 4 | Test of Darwin's naturalisation hypothesis. Comparison of the phylogenetic relatedness of superinvasives and other non-natives to the native species pools across different spatial scales based on TDWG biomes (Biodiversity Information Standards Taxonomic Databases Working Group). a, Phylogenetic relatedness calculated for level 2 of TDWG biomes corresponding to the spatial extent of regions (subcontinents) (n = 45 subcontinents, 13,218 grid cells). **b,** Phylogenetic relatedness calculated for level 3 of TDWG biomes corresponding to "botanical countries" (which often ignore purely political considerations) (n = 270 botanical countries, 13,218 grid cells). **c,** Phylogenetic relatedness calculated for plants at level 4 of TDWG biomes corresponding to "basic recording units" where political integrity is recognized (n = 423 basic recording units, 13,218 grid cells). Results show standard effect size of mean phylogenetic distance estimated from 1000 randomizations. Significance was assessed as the lack of overlap between the 95% confidence interval and zero. An overlap on the other hand indicates non significance but there might be some exceptions. Temp Temperate, Trop Tropical, Au Australasia, Eu Europe, N Am North America, Pac Pacific, S Am South America. The bottom and top of boxes show the first and third quartiles respectively, the median is indicated by the horizontal line, the range of the data by the whiskers. Source data are provided as a Source Data file.

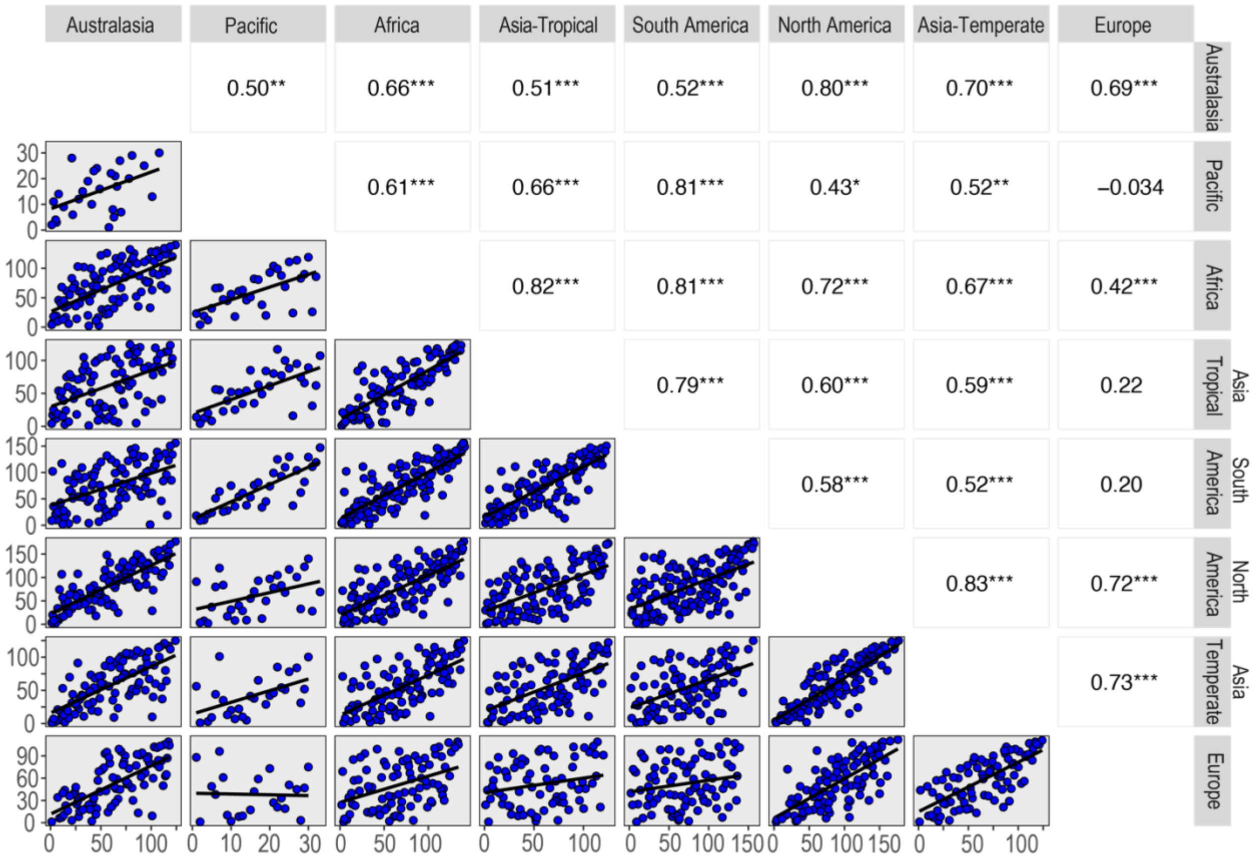

Supplementary Figure 5 | Comparison of ranks for plant families containing non-native species interchanged across continents. These plots show how the relative rank of proportional representation of non-natives from each plant family compares across continents. Each point in the lower half of the graph represents the ranking of plant families within a continent (*x* and *y*-axes) based the proportional representation of non-natives from that family relative to its total global diversity. The Spearman rank correlation coefficients between regions are presented in the top right. High correlations indicate that the non-native species in two regions are representatives of the same families. Spearman rank correlation test; P-values: **<0.05; ***<0.01

Supplementary Table 1: Bioclimatic variables included in the species distribution modeling of plants of the world.

Variable	Description
BIO1	Annual Mean Temperature
BIO2	Mean Diurnal Range (Mean of monthly (max temp – min temp))
BIO3	Isothermality (BIO2/BIO7) (* 100)
BIO4	Temperature Seasonality (standard deviation *100)
BIO5	Max Temperature of Warmest Month
BIO6	Min Temperature of Coldest Month
BIO7	Temperature Annual Range (BIO5-BIO6)
BIO8	Mean Temperature of Wettest Quarter
BIO9	Mean Temperature of Driest Quarter
BIO10	Mean Temperature of Warmest Quarter
BIO11	Mean Temperature of Coldest Quarter
BIO12	Annual Precipitation
BIO13	Precipitation of Wettest Month
BIO14	Precipitation of Driest Month
BIO15	Precipitation Seasonality (Coefficient of Variation)
BIO16	Precipitation of Wettest Quarter
BIO17	Precipitation of Driest Quarter
BIO18	Precipitation of Warmest Quarter
BIO19	Precipitation of Coldest Quarter

Supplementary Table 2 | Edges specifications for network analysis of regional donors and recipients of non-native species and phylogenetic diversity across continents. PD_{ses} represent phylogenetic exchange after correcting for species richness. ATEMP Asia–Temperate, ATROP Asia–Tropical, AUS Australasia, NAMER North America, SAMER South America.

Donor	PD	SR	PD_{ses}	Recipient	SR received	PD received	PD_{ses} received
AFRICA	28574.8	587	-3.0	SAMER	1653	54994.6	38092.0
ATEMP	26639.1	488	-1.2	SAMER	1653	54994.6	38092.0
ATROP	28950.0	588	-2.5	SAMER	1653	54994.6	38092.0
AUS	18668.4	268	-0.8	SAMER	1653	54994.6	38092.0
EUROPE	15946.4	244	-3.6	SAMER	1653	54994.6	38092.0
NAMER	35124.4	824	-3.2	SAMER	1653	54994.6	38092.0
PACIFIC	3645.3	34	-3.9	SAMER	1653	54994.6	38092.0
AFRICA	19991.0	305	0.3	ATEMP	1223	43823.2	23616.7
ATROP	27189.9	500	1.3	ATEMP	1223	43823.2	23616.7
AUS	13555.2	170	-0.8	ATEMP	1223	43823.2	23616.7
EUROPE	20199.8	452	-7.7	ATEMP	1223	43823.2	23616.7
NAMER	21899.2	404	-2.4	ATEMP	1223	43823.2	23616.7
PACIFIC	2034.8	14	-2.6	ATEMP	1223	43823.2	23616.7
SAMER	19136.7	319	-2.0	ATEMP	1223	43823.2	23616.7
AFRICA	29495.7	626	-4.3	NAMER	2521	71978.9	39124.7
ATEMP	40255.5	1006	-3.6	NAMER	2521	71978.9	39124.7
ATROP	30920.1	611	-1.7	NAMER	2521	71978.9	39124.7
AUS	21694.0	355	-2.4	NAMER	2521	71978.9	39124.7
EUROPE	29170.3	760	-10.4	NAMER	2521	71978.9	39124.7
PACIFIC	3041.7	30	-4.7	NAMER	2521	71978.9	39124.7
SAMER	37572.6	968	-6.1	NAMER	2521	71978.9	39124.7
AFRICA	20288.2	344	-3.0	ATROP	1029	41592.7	22856.8
ATEMP	26603.6	477	-0.4	ATROP	1029	41592.7	22856.8
AUS	13863.4	179	-1.7	ATROP	1029	41592.7	22856.8
EUROPE	9675.6	112	-2.6	ATROP	1029	41592.7	22856.8
NAMER	20176.9	345	-3.3	ATROP	1029	41592.7	22856.8
PACIFIC	3217.3	29	-3.9	ATROP	1029	41592.7	22856.8
SAMER	23499.0	451	-4.1	ATROP	1029	41592.7	22856.8

AFRICA	33316.2	890	-6.3	AUS	2078	60806.3	32413.1
ATEMP	30462.8	682	-2.6	AUS	2078	60806.3	32413.1
ATROP	23814.7	454	-2.5	AUS	2078	60806.3	32413.1
EUROPE	26015.7	634	-7.2	AUS	2078	60806.3	32413.1
NAMER	26928.1	551	-2.4	AUS	2078	60806.3	32413.1
PACIFIC	2937.0	33	-5.6	AUS	2078	60806.3	32413.1
SAMER	27594.6	611	-3.9	AUS	2078	60806.3	32413.1
AFRICA	22503.1	512	-5.5	EUROPE	2092	56442.2	29138.1
ATEMP	38607.8	1221	-5.9	EUROPE	2092	56442.2	29138.1
ATROP	18750.9	319	-1.9	EUROPE	2092	56442.2	29138.1
AUS	15974.1	258	-2.8	EUROPE	2092	56442.2	29138.1
NAMER	28177.4	638	-1.8	EUROPE	2092	56442.2	29138.1
PACIFIC	1317.8	8	-2.1	EUROPE	2092	56442.2	29138.1
SAME	17322.8	294	-2.8	EUROPE	2092	56442.2	29138.1
ATEMP	29233.7	587	-1.2	AFRICA	1545	51996.2	31417.1
ATROP	30666.8	637	-1.2	AFRICA	1545	51996.2	31417.1
AU	20868.2	327	-0.6	AFRICA	1545	51996.2	31417.1
EUROPE	16398.4	290	-5.5	AFRICA	1545	51996.2	31417.1
NAMER	26039.8	521	-3.0	AFRICA	1545	51996.2	31417.1
PACIFIC	4278.1	44	-4.5	AFRICA	1545	51996.2	31417.1
SAMER	31149.9	712	-3.9	AFRICA	1545	51996.2	31417.1
AFRICA	9725.7	103	-0.1	PACIFIC	280	17889.8	8798.6
ATEMP	9737.3	106	-0.5	PACIFIC	280	17889.8	8798.6
ATROP	13074.7	167	-0.3	PACIFIC	280	17889.8	8798.6
AUS	8972.8	93	-0.5	PACIFIC	280	17889.8	8798.6
EUROPE	4281.7	28	0.2	PACIFIC	280	17889.8	8798.6
NAMER	10025.5	106	0.1	PACIFIC	280	17889.8	8798.6
SAMER	11482.8	131	0.3	PACIFIC	280	17889.8	8798.6

Supplementary Table 3 | Nodes specifications of network analysis. Spread of the total numbers of non-native species (SR), phylogenetic diversity (PD), and phylogenetic diversity correcting for species richness (PD_{ses}) per continental region. ATEMP Asia–Temperate, ATROP Asia–Tropical, AUS Australasia, NAMER North America, SAMER South America.

Continent	SR donated	PD donated	PD_{ses} donated	Latitude	Longitude
SAMER	1651	52360.9	80129.9	-13.929539	-61.653497
ATEMP	2436	66352.6	211691.4	50.2116418	95.4993249
NAMER	1732	56200.4	214194.7	57.2152388	-92.489215
ATROP	1527	53214.9	160731.1	14.8164716	106.621216
AUS	711	33001.8	191837.4	-25.482123	132.485806
EUROPE	1387	41464.3	318760.2	51.7884163	5.23062231
AFRICA	1666	50404.2	124399.6	6.3810789	18.2453334
PACIFIC	60	4705.5	1084217.4	-17.8629	177.902602

Supplementary Method

GreenMaps: a Tool for Addressing the Wallacean Shortfall in the Global Distribution of Plants

– ODMAP Protocol –

Barnabas H. Daru

2021-04-02

Overview

Authorship

Contact : barnabas.daru@tamucc.edu

Study link: 10.1101/2020.02.21.960161

Model objective

Model objective: Mapping and interpolation

Target output: continuous occurrence probabilities and binary maps of potential presence for each species

Focal Taxon

Focal Taxon: Vascular plants

Location

Location: global, excluding Antarctica

Scale of Analysis

Spatial extent: -180, 180, -59.47308, 83.57031 (xmin, xmax, ymin, ymax)

Spatial resolution: 4.625 km × 4.625 km

Temporal extent: Contemporary

Temporal resolution: N/A

Boundary: natural

Biodiversity data

Observation type: citizen science

Response data type: presence-only

Predictors

Predictor types: climatic, topographic

Hypotheses

Hypotheses: Raw plant occurrence data alone should not be used indiscriminately due to inherent sampling biases, impediments that contribute to Wallacean shortfall (i.e. the paucity of species' geographic information). Species distribution models (SDMs) provide an unbiased and easily interpretable estimate of improving representativeness and coverage of plant species distributions. This assumes that plant species are affected by key physical and biological attributes such as precipitation, temperature, primary productivity, and elevation.

Assumptions

Model assumptions: 1) Relevant ecological drivers (or proxies) of species distributions are included. 2) Species are at equilibrium with their environment. 3) Sampling is adequate and representative (and any biases are accounted for/corrected).

Algorithms

Modelling techniques: Species distribution modeling were built using 4 modelling algorithms (generalised linear models, GLMs, generalised boosted models, GBMs, random forests, RFs, and maximum entropy, MaxEnt).

Model complexity: N/A

Model averaging: The four modeling algorithms were combined to generate an ensemble SDM predictions.

Workflow

Model workflow: Prior to model building, all predictor variables were standardised. In each model, I only included the most important and weakly correlated variables. Univariate variable importance for each predictor was assessed in a 5-fold spatial block cross-validation design. Ensemble predictions from SDMs and richness models were derived using ensemble means. The averaged ROC AUC scores were used as model predictive performance measures, following a 5-fold spatial block cross-validation.

Software

Software: R version 4.0.4 (2021-02-15) – "Lost Library Book" with packages dismo, raster, phyloregion, randomForest

Code availability: https://darunabas.github.io/phyloregion/index.html

Data availability: Dryad data repository: (https://datadryad.org/stash/share/UFhi3ts7G6sIjHj1IanUUK1V8AVzh4ep6hEUdqCJV9k)

Data

Biodiversity data

Taxon names: Phylum Tracheophyta

Taxonomic reference system: I follow the taxonomy of World Flora Online (www.worldfloraonline.org)

Ecological level: species

Data sources: Occurrence records were obtained from a variety of sources, including herbarium specimens, primary literature, personal observation, and online data repositories including iNaturalist, the Global Biodiversity Information Facility (https://www.gbif.org/), Integrated Digitized Biocollections (www.idigbio.org), and Botanical Information and Ecology Network. These occurrence records were obtained for a total of 230,000 species represented within 382 families.

Sampling design: The occurrence records include a mix of data from vouchered specimens that can be linked to a tangible material in a museum or herbarium, as well as opportunistic observations and monitoring data from citizen science programs.

Sample size: Variable

Clipping: Two stringent spatial filters were employed to restrict species' distributions to their known native ranges (i.e., realized niches) and to prevent erroneous records and predictions in areas that contain suitable habitat but are unoccupied by the species (i.e., fundamental niche). First, I applied the spatial constraint, APGfamilyGeo, which are expert drawn occurrence polygons ("expert maps") of plant family distributions to restrict species to within these distributions under the assumption that a species' native range should not extend beyond its family's distribution. Second, I applied GeoEigenvectors, which are orthogonal variables representing spatial relationships among cells in a grid, encompassing the geometry of the study region at various scales. For the latter, I generated a pairwise geographical connectivity matrix among grid cells to establish a truncation distance for the eigenvector-based spatial filtering, returning a total of 150 spatial filters. These filters were then resampled to the same resolution as the input environmental variables, and I included the first 14 spatial filters along with the 19 bioclimatic variables in the species distribution modeling.

Scaling: These records were thoroughly cleaned to reconcile names to follow currently accepted taxonomies [e.g., World Flora Online (www.worldfloraonline.org)], and to remove duplicates and records with doubtful or imprecise localities.

Cleaning: After data cleaning, I only considered species with at least 20 unique presences. However, I accounted for species with fewer than the threshold number of records for SDMs by using the bioclim model in the R package dismo to generate initial predictions as additional input occurrence points for downstream modeling. I set the threshold at 0.5 and then used the *randomPoints* function in the R package *dismo* to sample random points based on the suitability model output from the bioclim model. I set the *prob* argument in the *randomPoints* function to TRUE, meaning that the values in mask are interpreted as probability weights such that cells with higher suitability will have more probability to be selected as pseudo-presences. These

pseudo-points were used in addition to the cleaned dataset as inputs for the species distribution modelling.

Absence data: None

Background data: Species distributions were modelled using 10,000 background points

Data partitioning

Training data: All data were used for model training and model performance was only assessed internally.

Validation data: N/A

Predictor variables

Predictor variables: Annual Mean Temperature, Mean Diurnal Range, Isothermality, Temperature Seasonality, Max Temperature of Warmest Month, Min Temperature of Coldest Month, Temperature Annual Range, Mean Temperature of Wettest Quarter, Mean Temperature of Driest Quarter, Mean Temperature of Warmest Quarter, Mean Temperature of Coldest Quarter, Annual Precipitation, Precipitation of Wettest Month, Precipitation of Driest Month, Precipitation Seasonality, Precipitation of Wettest Quarter, Precipitation of Driest Quarter, Precipitation of Warmest Quarter, Precipitation of Coldest Quarter, and Elevation.

I also applied GeoEigenvectors, which are orthogonal variables representing spatial relationships among cells in a grid, encompassing the geometry of the study region. Thus, I generated a pairwise geographical connectivity matrix among grid cells to establish a truncation distance for the eigenvector-based spatial filtering, returning a total of 150 spatial filters. These filters were then resampled to the same resolution as the input environmental variables, and were included with the bioclimatic variables in the species distribution modeling.

Data sources: Climate: Bioclimatic variables were derived from WorldClim version 2 (https://www.worldclim.org/) for a total of 19 variables and elevation. These variables are available as GeoTiff (.tif) files at a resolution of 4.625 km × 4.625 km.

Spatial extent: -180, 180, -60, 90 (xmin, xmax, ymin, ymax)

Spatial resolution: The raw resolution of the climate data was 2.5 minutes.

Coordinate reference system: WGS 1984, EPSG:4326

Temporal extent: 1970-2000

Transfer data

Data sources: Not applicable

Spatial resolution: Not applicable

Temporal extent: Not applicable

Temporal resolution: Not applicable

Models and scenarios: Not applicable

Data processing: Not applicable

Quantification of Novelty: Not applicable

Model

Variable pre-selection

Variable pre-selection: Predictors were pre-selected based on their hypothesised ecological relevance for the distribution of plant species.

Multicollinearity

Multicollinearity: Principal components analysis was used for dimension reduction prior to modelling. This reduces collinearity greatly or eliminates it entirely.

Model settings

Model settings (extrapolation):

1) Generalised linear models (GLMs): GLMs were generated assuming a logistic link function and a binomial error distribution of the response. Linear, quadratic and polynomial terms (second and third order) of each climatic predictor were included in the initial models, and a stepwise procedure using the AIC criterion was used to select the most significant terms.

2) Generalised Boosted Models (GBMs): GBMs were fitted with an interaction depth of 4, a learning rate of 0.001, and a maximum of 5000 trees fitted to the data.

3) Random Forests (RFs): the number of trees grown were set to 500 and the number of predictors to be chosen randomly at each node were set to (total number of predictors – 1)

4) Maximum entropy (MaxEnt): I enabled the use of all six feature classes (linear, product, quadratic, hinge, threshold and categorical) for modelling species responses to environmental variables. The default value of 1.0 was used as the regularization parameter, which affects how closely the training data is fitted.

Model estimates

Variable importance: Covariate importance calculated as averaged model drop contributions to identify variables not contributing importantly to model robustness (explained deviance loss when variable dropped).

Model selection - model averaging - ensembles

Model selection: I calculated the mean probability of occurrence from all four modelling techniques as a consensus method for combining the output of different single-models.

Analysis and Correction of non-independence

Spatial autocorrelation: Potential non-independence in the data was not accounted for in the models

Threshold selection

Threshold selection: Continuous probabilities were converted into binary ‘presence–absence’ predictions using the threshold selection method based on maximising the sum of sensitivity and specificity. I considered areas with a habitat suitability above the threshold as ‘presence’ and those below as ‘absence’.

Assessment

Performance statistics

Performance on training data: Model performance was evaluated using area under curve (AUC) of the receiver operating characteristic (ROC)

Performance on validation data: AUC

Performance on test data: AUC

Plausibility check

Response shapes: Maps of modelled predictions were checked by experts for an ad-hoc subset of species.

Expert judgement: Expert judgements, e.g., map display

Prediction

Prediction output

Prediction unit: presence and absence

Post-processing: The final outputs consisted of a modeled range map stored in raster GeoTiff (.tif) format at grid cell resolution of 0.5 degree equivalent to 50 km at the equator. In addition, I also stored the input occurrence points in CSV format for each species along with information on whether random points were added for species with too few records.

Uncertainty quantification

Algorithmic uncertainty: Model-based uncertainty was accounted for in the predictions by calculating the mean probability of occurrence from all four modelling techniques as a consensus method for combining the output of different single-models.

Input data uncertainty: To improve the utility of species with too few records for SDMs, buffers of 1-2 km radius were generated as spatial offsets in Poisson point process model around each point and randomly (Poisson) distributed points (adding up to 20) within the boundaries of the buffers (a good SDM requires 10-200 unique records)

Parameter uncertainty: N/A

Scenario uncertainty: N/A

Novel environments: The results are visualized using maps in geographic space.
