# Supplementary material for: Widespread homogenization of plant communities in the Anthropocene
Source: Nat Commun. 2021 Dec 6;12:6983. doi: 10.1038/s41467-021-27186-8 (PMC8648934; doi:10.1038/s41467-021-27186-8)
Supplement: Supplementary file 3 — Reporting Summary [file 41467_2021_27186_MOESM3_ESM.pdf]

nature research

Corresponding author(s): BARNABAS H. DARU

Last updated by author(s): Oct 31, 2021

Reporting Summary

Nature Research wishes to improve the reproducibility of the work that we publish. This form provides structure for consistency and transparency in reporting. For further information on Nature Research policies, see our Editorial Policies and the Editorial Policy Checklist.

Statistics

For all statistical analyses, confirm that the following items are present in the figure legend, table legend, main text, or Methods section.

n/a	Confirmed	
☐	☒	The exact sample size (*n*) for each experimental group/condition, given as a discrete number and unit of measurement
☐	☒	A statement on whether measurements were taken from distinct samples or whether the same sample was measured repeatedly
☒	☐	The statistical test(s) used AND whether they are one- or two-sided<br>*Only common tests should be described solely by name; describe more complex techniques in the Methods section.*
☐	☒	A description of all covariates tested
☐	☒	A description of any assumptions or corrections, such as tests of normality and adjustment for multiple comparisons
☐	☒	A full description of the statistical parameters including central tendency (e.g. means) or other basic estimates (e.g. regression coefficient) AND variation (e.g. standard deviation) or associated estimates of uncertainty (e.g. confidence intervals)
☐	☒	For null hypothesis testing, the test statistic (e.g. *F*, *t*, *r*) with confidence intervals, effect sizes, degrees of freedom and *P* value noted<br>*Give P values as exact values whenever suitable.*
☒	☐	For Bayesian analysis, information on the choice of priors and Markov chain Monte Carlo settings
☒	☐	For hierarchical and complex designs, identification of the appropriate level for tests and full reporting of outcomes
☒	☐	Estimates of effect sizes (e.g. Cohen's *d*, Pearson's *r*), indicating how they were calculated

Our web collection on statistics for biologists contains articles on many of the points above.

Software and code

Policy information about availability of computer code

Data collection	phyloregion version 1.0.4 (an R package)
Data analysis	Data was analyzed using R package phyloregion version 1.0.7, raster version 3.4-13, nlme version 3.1-152, Gephi version 0.9.2

For manuscripts utilizing custom algorithms or software that are central to the research but not yet described in published literature, software must be made available to editors and reviewers. We strongly encourage code deposition in a community repository (e.g. GitHub). See the Nature Research guidelines for submitting code & software for further information.

Data

Policy information about availability of data

All manuscripts must include a data availability statement. This statement should provide the following information, where applicable:

- Accession codes, unique identifiers, or web links for publicly available datasets
- A list of figures that have associated raw data
- A description of any restrictions on data availability

The plant species range maps included in this study come from a newly developed species database called GreenMaps (https://doi.org/10.1101/2020.02.21.960161). GreenMaps includes global distribution maps for ~230,000 vascular plant species. The maps were generated using species distribution models derived from carefully curated species occurrence records, and the dataset is archived on Dryad (https://datadryad.org/stash/share/UFhi3ts7G6sIjHj1IanUUK1V8AVzh4ep6hEUdqCJV9k). Occurrence records were obtained from a variety of sources, including herbarium specimens, primary literature, personal observation, and online data repositories including the Global Biodiversity Information Facility (Accession: https://doi.org/10.15468/dl.7ujp48; https://doi.org/10.15468/dl.jw4u5a, and https://doi.org/10.15468/dl.m8dzn5), and Integrated Digitized Biocollections (https://www.idigbio.org/). The phylogeny used for the analyses is a published phylogeny that is already available in public repositories. Specifically, the plant phylogeny was downloaded from Smith and

Brown (https://doi.org/10.1002/ajb2.1019). Source data are provided with this paper.

Field-specific reporting

Please select the one below that is the best fit for your research. If you are not sure, read the appropriate sections before making your selection.

☐ Life sciences ☐ Behavioural & social sciences ☒ Ecological, evolutionary & environmental sciences

For a reference copy of the document with all sections, see nature.com/documents/nr-reporting-summary-flat.pdf

Ecological, evolutionary & environmental sciences study design

All studies must disclose on these points even when the disclosure is negative.

Study description	Using plant distributions from the newly developed species database, GreenMaps, in combination with comprehensive published phylogeny, we quantify how species introductions and recent extinctions have impacted local (α) and between community (β) plant diversity across spatial scales. We then explore differences in biotic homogenization under varying future scenarios of increasing extinction intensity. We map the distribution of each species using distribution models fitted to carefully curated species occurrence records, and contrast 'Holocene' and 'Anthropocene' species diversities around the globe. We define species composition in the Holocene as the native species' assemblages in each region before widespread migration by humans as initiated by the Columbian Exchange circa 1492. We quantify changes in plant community diversity (α-diversity) between the Holocene and Anthropocene epochs, and examine the signature of increasing homogenization (lower β-diversity) at regional and global scales. We then evaluate the relative contribution of introductions vs extirpations in restructuring global plant diversity, and the macroecological correlates of changes in floristic composition.
Research sample	We used the newly developed species database, GreenMaps, to estimate native plant species' distributions. GreenMaps includes global distribution maps for ~230,000 vascular plant species. Because this is a global study, the rationale for the choice of sample size is to aim to sample all the vascular plant species of the world (estimated at 300,000 species). Maps were generated using species distribution models – the statistical estimation of species geographic distributions based on only some known occurrences and environmental conditions – derived from carefully curated species occurrence records. Occurrence records were obtained from a variety of sources, including herbarium specimens, primary literature, personal observation, and online data repositories including the Global Biodiversity Information Facility, and Integrated Digitized Biocollections (https://www.idigbio.org/). These records were thoroughly cleaned to reconcile names to follow currently accepted taxonomies [e.g., World Flora Online (www.worldfloraonline.org)], and to remove duplicates and records with doubtful or imprecise localities. Two stringent spatial filters were employed to restrict species' distributions to their known native ranges (i.e., realized niches) and to prevent erroneous records and predictions in areas that contain suitable habitat but are unoccupied by the species (i.e., fundamental niche). First, we applied the spatial constraint, APGfamilyGeo, which are expert drawn occurrence polygons ("expert maps") of plant family distributions (see Data availability) to restrict species to within these distributions. Second, we applied GeoEigenvectors, which are orthogonal variables representing spatial relationships among cells in a grid, encompassing the geometry of the study region at various scales. For the latter, we generated a pairwise geographical connectivity matrix among grid cells to establish a truncation distance for the eigenvector-based spatial filtering, returning a total of 150 spatial filters. These filters were then resampled to the same resolution as the input environmental variables, and were included with the bioclimatic variables in the species distribution modeling. Bioclimatic variables were derived from WorldClim for a total of 19 variables (Supplementary Table 1). Species distribution models (SDMs) were fitted using four different algorithms: generalized linear models (GLM), generalized boosted models (GBM), maximum entropy (MaxEnt), and random forests (RF) with a binomial error distribution (with logit link). Model settings were chosen to yield intermediately complex response surfaces. Model performance was evaluated using area under the receiver operating curve (AUC) and true skill statistic (TSS) scores. AUC scores range from 0 to 1 and should be maximized whereas TSS scores range from -1 to 1. Prior to model building, all predictor variables were standardized. Univariate variable importance for each predictor was assessed in a 5-fold spatial block cross-validation design. The ensemble predictions from species distribution models were derived using un-weighted ensemble means. Predictive model performance was assessed using a 5-fold spatial block cross-validation. We generated a total of 230,000 range maps, representing species within 382 families at a resolution of 50 × 50 km which was also resampled to 100 × 100 km, making it the largest and only global assessment of geographic distributions for plants at the species-level. Our approach of modeling species distributions follows the guidelines of ODMAP (Overview, Data, Model, Assessment, Prediction), a comprehensive framework of best practices for reporting species distribution models. These maps were stacked and converted to a community matrix for downstream analyses. We also provide a new R function, sdm, for performing the SDMs across four algorithms (random forest, generalized linear models, gradient boosted machines, and MaxEnt) tailored for SDMs of large datasets. The sdm function is included in our R package phyloregion along with improved documentation and vignettes to show practical application of this functionality under various modelling scenarios. The sdm function was designed with multiple checks such that any species that did not meet one or more checks were filtered out. A feature of novelty of the sdm function is the addition of an algorithm that allows a user to exclude records that occur within a certain distance to herbaria, museums or other infrastructure. By default, we used the most updated version of Index Herbariorum, a global directory of herbaria, but a user has the option to specify their own infrastructure to exclude.<br>We validated the output distribution maps against the Kew Plants of the World Online database (POWO; http://www.plantsoftheworldonline.org/), which includes native distribution maps for all plants of the world within major biogeographically defined areas recognized by the Biodiversity Information Standards (also known as the Taxonomic Databases Working Group (TDWG)). Although the Kew's distributions of native species are largely based on state/province level such that if a species was observed in any location within a state the whole state is marked as its distribution range, our GreenMaps approach only used the Kew distributions to restrict modeled species distributions within such biogeographic areas. The range map rasters were converted to a community matrix using the function raster2comm in our new R package phyloregion for downstream analysis.<br>The full workflow is described in Daru (2020; https://doi.org/10.1101/2020.02.21.960161). The range map rasters were converted to a community matrix using the function raster2comm in our new R package phyloregion (Daru et al. 2020; https://doi.org/10.1111/2041-210X.13478) and available on DRYAD at https://datadryad.org/stash/share/

UFhi3ts7G6sIjHj1IanUUK1V8AVzh4ep6hEUdqCJV9k

Sampling strategy	Given the scale of of this study being global study, the sampling strategy was to aim to sample all the vascular plant species of the world (estimated at 300,000 species). We were only able to find ~230,000 species (of 300,000 estimated species).
Data collection	Data was obtained by B.H.D. from the GreenMaps database (Daru 2020; https://doi.org/10.1101/2020.02.21.960161) using R version 4.1.0. The phylogenies were obtained by B.H.D. from Smith & Brown (https://github.com/FePhyFoFum/big_seed_plant_trees) using R version 4.1.0
Timing and spatial scale	The spatial scale is global. The analyses also vary along spatial grains and extent.<br>For spatial grains, the reported results were analyzed at a spatial resolution of 100 × 100 km. We repeated all analyses at spatial grid resolution of 50 × 50 km.<br>In terms of spatial extent, we run the analyses at a global extent. We then performed sensitivity analyses at various extents varying along continental, national to provincial levels.<br>Because plant occurrence records are constantly being mobilized online, we strive to use the most updated version as possible. Thus the timing of our data collection is from 2020-2021.
Data exclusions	We did not exclude any data.
Reproducibility	We share all scripts and code necessary to repeat the analyses described in this study in a new R package phyloregion (https://CRAN.R-project.org/package=phyloregion). In addition, all data necessary to repeat the analyses described here have been made available through the Dryad digital data repository (https://datadryad.org/stash/share/UFhi3ts7G6sIjHj1IanUUK1V8AVzh4ep6hEUdqCJV9k).
Randomization	To understand temporal changes in alpha diversity across plant communities, we assessed changes in phylogenetic (α) diversity standardized for species richness by calculating standard effects sizes of phylogenetic diversity in communities by shuffling the tips in the phylogeny based on 1000 randomizations. For each iteration of the randomization, the analysis was regenerated using the same set of spatial conditions, but using the randomized version of the tree after which the z-score for each index value was calculated (observed - expected / sqrt (variance)).<br>We also evaluated whether introduced plant species were more likely to have become naturalized in recipient communities in the absence of close relatives—Darwin's naturalization hypothesis—by comparing the mean phylogenetic distance between each non-native species and its nearest phylogenetic neighbor in the recipient flora. Larger mean phylogenetic distances indicate that non-native species tend to be less closely related to the native flora. Significance was assessed by comparing the distribution of observed phylogenetic distances to a null model shuffling non-native status randomly on the tips of the phylogeny (1000 replicates) as implemented in the R package phyloregion (Daru et al. 2020).
Blinding	Blinding was not relevant to the study, as there were no experimental treatments.

Did the study involve field work? ☐ Yes ☒ No

Reporting for specific materials, systems and methods

We require information from authors about some types of materials, experimental systems and methods used in many studies. Here, indicate whether each material, system or method listed is relevant to your study. If you are not sure if a list item applies to your research, read the appropriate section before selecting a response.

Materials & experimental systems

n/a	Involved in the study
☒	☐ Antibodies
☒	☐ Eukaryotic cell lines
☒	☐ Palaeontology and archaeology
☒	☐ Animals and other organisms
☒	☐ Human research participants
☒	☐ Clinical data
☒	☐ Dual use research of concern

Methods

n/a	Involved in the study
☒	☐ ChIP-seq
☒	☐ Flow cytometry
☒	☐ MRI-based neuroimaging
